# Comparative performance of transcriptome assembly methods for non-model organisms

**DOI:** 10.1186/s12864-016-2923-8

**Published:** 2016-07-27

**Authors:** Xin Huang, Xiao-Guang Chen, Peter A. Armbruster

**Affiliations:** 1 Department of Biology, Georgetown University, 37th and O Streets NW, Washington, DC, 20057 USA; 2 Key Laboratory of Prevention and Control for Emerging Infectious Diseases of Guangdong Higher Institutes, Department of Pathogen Biology, School of Public Health and Tropical Medicine, Southern Medical University, Guangzhou, China

**Keywords:** Transcriptome assembly, Non-model organisms, *De novo* assembly, Reference-based re-assembly, TransPS, Genome-guided assembly, Next-generation sequencing, *Aedes albopictus*

## Abstract

**Background:**

The technological revolution in next-generation sequencing has brought unprecedented opportunities to study any organism of interest at the genomic or transcriptomic level. Transcriptome assembly is a crucial first step for studying the molecular basis of phenotypes of interest using RNA-Sequencing (RNA-Seq). However, the optimal strategy for assembling vast amounts of short RNA-Seq reads remains unresolved, especially for organisms without a sequenced genome. This study compared four transcriptome assembly methods, including a widely used *de novo* assembler (Trinity), two transcriptome re-assembly strategies utilizing proteomic and genomic resources from closely related species (reference-based re-assembly and TransPS) and a genome-guided assembler (Cufflinks).

**Results:**

These four assembly strategies were compared using a comprehensive transcriptomic database of *Aedes albopictus*, for which a genome sequence has recently been completed. The quality of the various assemblies was assessed by the number of contigs generated, contig length distribution, percent paired-end read mapping, and gene model representation via BLASTX. Our results reveal that *de novo* assembly generates a similar number of gene models relative to genome-guided assembly with a fragmented reference, but produces the highest level of redundancy and requires the most computational power. Using a closely related reference genome to guide transcriptome assembly can generate biased contig sequences. Increasing the number of reads used in the transcriptome assembly tends to increase the redundancy within the assembly and decrease both median contig length and percent identity between contigs and reference protein sequences.

**Conclusions:**

This study provides general guidance for transcriptome assembly of RNA-Seq data from organisms with or without a sequenced genome. The optimal transcriptome assembly strategy will depend upon the subsequent downstream analyses. However, our results emphasize the efficacy of *de novo* assembly, which can be as effective as genome-guided assembly when the reference genome assembly is fragmented. If a genome assembly and sufficient computational resources are available, it can be beneficial to combine *de novo* and genome-guided assemblies. Caution should be taken when using a closely related reference genome to guide transcriptome assembly. The quantity of read pairs used in the transcriptome assembly does not necessarily correlate with the quality of the assembly.

**Electronic supplementary material:**

The online version of this article (doi:10.1186/s12864-016-2923-8) contains supplementary material, which is available to authorized users.

## Background

The use of next-generation sequencing (NGS) technologies has been increasing dramatically over the past decade [1]. Due to the technological revolution in NGS, vast amounts of both transcriptome and genome sequences across a wide range of species are accumulating, especially from large-scale projects including Genome 10 K [2] and Insect 5 K [3]. Traditionally, model organisms have been chosen largely based on the ease with which they can be reared in the laboratory and used for genetic studies, or their evolutionary relatedness to human. However, in the current “-omics” era, a much greater variety of organisms can be studied at the genomic and transcriptomic level. This revolution in DNA sequencing technologies has far-reaching applications for the field of biology, dramatically increasing opportunities to elucidate gene regulatory networks [4] and the genetic basis of complex traits [5, 6]. Most importantly, NGS allows scientists to investigate biological questions at an unprecedented scale. An exceptional example is a phylogenomic analysis of the origin and diversification of major insect lineages using transcriptome sequencing and genomic data [7]. In this study, 103 species from all 33 extant insect orders have been sequenced. Combining genomic data from 41 arthropod species with transcriptomic data from 103 species, the analysis was able to resolve with high confidence the timing of the origin and diversification (topology) of insects [7], addressing a fundamental question regarding the history of life on Earth.

RNA-Sequencing (RNA-Seq) is one important NGS technology [8]. RNA-Seq samples the entire transcriptome in great depth under a particular experimental condition. The transcriptome includes all expressed sequences, which is a reduced representation of the genome. RNA-Seq has many exciting applications [8, 9], including the phylogenomic example mentioned above, single nucleotide polymorphism (SNP) discovery, small RNA profiling, novel transcript or splice variant discovery and comparison of global transcriptional mRNA profiles under distinct environmental conditions, e.g., benign vs. ecologically stressful conditions. RNA-Seq is a powerful tool to advance these applications for at least three reasons. First, RNA-Seq is able to capture the expression of (ideally) all genes under the specific experimental conditions. Second, it does not require any prior genetic information, which is well suited for non-model organisms. Third, RNA-Seq is cost-effective and affordable for most laboratories. For nearly all applications of RNA-Seq, transcriptome assembly is challenging but a crucial first step for accurate downstream genetic analyses [10].

*De novo* transcriptome assembly programs, which assemble short RNA-Seq reads without a reference, are the default choice for organisms without a genome sequence. *De novo* assembly is particularly suitable for non-model organisms, but can also be a useful assembly strategy for organisms with a genome sequence. This is because *de novo* assembly programs are not constrained by alignments to a reference genome and can therefore discover novel transcripts and splice variants that are not annotated in the genome [10]. However, *de novo* assembly is usually memory intensive, and requires high sequence coverage compared with reference-based assembly [10]. In addition, *de novo* methods tend to produce fragmented assemblies. Genomic resources from closely related species, including genomic scaffolds and protein sequences, can serve as a reference to guide transcriptome assembly for non-model organisms and may lead to less fragmented assemblies. Therefore, transcriptome assembly for non-model organisms can potentially benefit from genomic resources of a closely related species, as previously demonstrated [11–14]. Due to the ever-expanding pool of organisms with a sequenced genome, many researchers studying non-model organisms without a genome sequence but with intriguing ecological, evolutionary and public health attributes will soon find closely related genomic resources available.

Without a genome sequence for the species of interest (true for most organisms), genomic resources (protein sequences and/or genomic scaffolds) from a closely related species, if available, can serve as reference scaffolds to re-align *de novo* assembled contigs. For this purpose, our laboratory has developed reference-based re-assembly [12, 13], which is based on Scaffolding using Translation Mapping (STM) [11]. STM aligns *de novo* assembled contigs to protein sequences from a closely related species, and subsequently re-assembles the contigs according to the alignment results [11]. STM has been demonstrated to reduce redundancy and improve contig length relative to *de novo* assembly [11]. Reference-based re-assembly is modified from STM as a two-step process to incorporate both protein sequences and genomic scaffolds from a closely related species, which utilizes not only the protein coding sequences, but also genomic sequences including 5′ and 3′ untranslated regions as well as other non-coding regions [12, 13]. Reference-based re-assembly has been shown to improve *de novo* assembly by increasing contig length and reducing redundancy [12, 13, 15]. Another similar approach to STM is Transcriptome Post-Scaffolding (TransPS) [14], which also utilizes protein sequences from a closely related species in order to re-assemble the *de novo* contigs according to how they align to the reference protein sequences. In contrast to reference-based re-assembly, TransPS merges contigs with non-overlapping alignments to the same reference protein [14]. However, TransPS does not utilize genomic sequences from a closely related species. TransPS reduces the number of contigs to as few as 10 % of the number of *de novo* assembled contigs, eliminating contigs that are redundant and increasing coverage for the reference protein sequences relative to the *de novo* assembly [14].

An alternative method that has been proposed to improve transcriptome assembly is genome-guided assembly, which utilizes the genome sequence of the same species as a reference to guide the transcriptome assembly [16]. If a high quality reference genome is available, this method usually generates longer contigs and is much less computationally demanding than *de novo* assembly [10]. With a well-annotated genome sequence, genome-guided assembly has been demonstrated to outperform *de novo* assembly [17]. If the genome assembly from the species of interest is not well annotated, and a well-annotated genome assembly from a closely related species is available, researchers may utilize the closely related genome to guide transcriptome assembly. However, few studies have evaluated the efficacy and accuracy of this approach (but see [18]).

The optimal strategy for transcriptome assembly will depend upon the downstream analysis and computational resources available. Data associated with extensive transcriptome sequencing in *Ae. albopictus* provide an outstanding opportunity to evaluate multiple methods of transcriptome assembly. Rich transcriptomic resources have been established across multiple life stages for *Ae. albopictus*, including developing embryos [12], pharate larvae [13, 19] and adults [15]. Therefore, the vast majority of all the expressed genes in the genome are included in these data. Furthermore, a closely related species, *Aedes aegypti*, has a well-annotated genome [20], which provides protein and genomic reference scaffolds for improving *de novo* transcriptome assembly with reference-based re-assembly and TransPS methods. In addition, a draft genome sequence for *Ae. albopictus* has recently been published [21], which allows for a genome-guided assembly. Finally, the more completely assembled and annotated *Ae. aegypti* genome assembly provides an excellent opportunity to evaluate the efficacy and accuracy of using a closely related reference genome to guide transcriptome assembly.

No study has thus far compared *de novo* assembly with both transcriptome re-assembly via protein reference scaffolding (i.e., reference-based re-assembly and TransPS) and genome-guided assembly simultaneously. This study utilized a comprehensive transcriptomic database of *Ae. albopictus* to investigate the optimal transcriptome assembly strategy, comparing four assembly methods: a) *de novo* assembly using Trinity [22, 23], b) reference-based re-assembly using proteomic and genomic resources from *Ae. aegypti*, c) TransPS [14] using the *Ae. aegypti* proteome and d) genome-guided assembly by Cufflinks [24] using the recently sequenced *Ae. albopictus* genome and the well-annotated *Ae. aegypti* genome. The quality of the various assemblies was assessed by the number of contigs generated, contig length distribution, percent paired-end read mapping, and gene model representation via BLASTX [25]. We also examined differences between the gene models identified by each assembly strategy to determine whether unique subsets of gene models were identified by each method. In addition, we examined how the amount of data (number of sequence reads) affected these quality metrics of the assembly. Results from this study are particularly relevant to non-model organisms with genomic resources from a closely related species. Furthermore, the genome assembly of *Ae. albopictus* is fragmented due in part to its large size (1.9 Gb) and high composition (68 %) of repetitive elements [21]. However, this assembly is comparable to other published insect genomes [3], and therefore results from this study can be extended to emerging model organisms with a more fragmented genome assembly compared to traditional model organisms.

## Methods

### RNA-Seq data

**Fig. 1:**
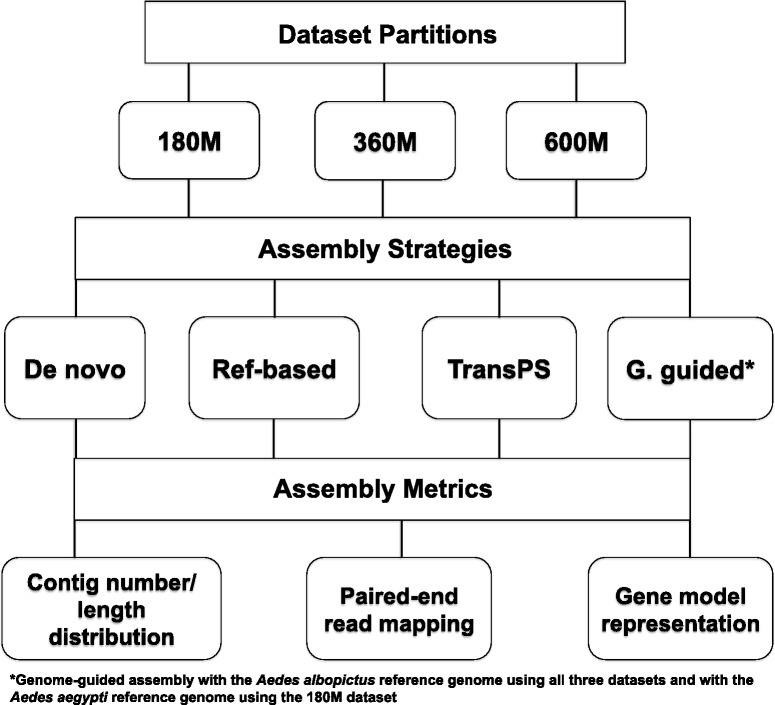
Flow chart for the experimental design of this study. Three datasets, 180 M, 360 M and 600 M, were used for each of the four assembly strategies described in the text. Subsequently, all fourteen assemblies were assessed by the metrics contig number and length distribution, percent paired-end reads mapped back to the assembly and gene model representation. De novo stands for *de novo* assembly, Ref-based for reference-based re-assembly, TransPS for transcriptome post-scaffolding, and G. guided for genome-guided assembly

Cleaned RNA-Seq paired-end reads from pooled tissue samples described in previous publications from this laboratory were used for this study. These data include reads from developing embryos [12], pharate larvae [13, 19] and adults [15] of *Ae. albopictus* under diapause and non-diapause conditions (read length = 101 bp). General procedures for generating these RNA-Seq reads from *Ae. albopictus* have been described previously [26]. Quality filtering of the RNA-Seq raw reads was consistent across studies and described in detail in previous publications from this laboratory [12, 13, 15]. 122,687,107 cleaned read pairs were obtained from the developing embryos, 289,860,821 cleaned read pairs were obtained from the pharate larvae and 182,036,362 cleaned read pairs were obtained from the adult stage. In order to investigate the effect of data quantity on the quality of the assembled transcriptome, read pairs from the three life stages (embryo, larva, adult) were partitioned into three datasets containing increasing number of read pairs. We included reads from all three life stages in each dataset to maximize representation of expressed genes in the analysis. The smallest dataset, termed 180 M, was comprised of 181,225,819 read pairs, taken from one biological replicate from all experimental treatments (diapause vs. non-diapause) in each life stage. In cases where the libraries had been sequenced on different runs, the largest libraries from one lane in each run were included. The amount of data in the 180 M dataset is the yield of approximately two lanes of Illumina HiSeq 2000 sequencing. The medium dataset, termed 360 M, was comprised of 344,799,731 read pairs, taken from the remaining biological replicates of each treatment and life stage. The amount of data in the 360 M dataset is the yield of approximately four lanes of Illumina HiSeq 2000 sequencing. The largest dataset, termed 600 M, was comprised of 594,584,290 read pairs, taken from all sequencing lanes and runs across all treatments and life stages. The amount of data in the 600 M dataset is the yield of approximately seven lanes of Illumina HiSeq 2000 sequencing. The transcriptome assembly strategies utilized in this study and described below were applied to all three datasets (Fig. 1).

### Transcriptome assembly strategies

#### *De novo* assembly

Trinity ([23]; version 20140717) was used for *de novo* assembly. Reads were *in silico* normalized with the option --normalize_max_read_cov 100 (based on recommendations in Haas et al. [23]). In addition, --min_kmer_cov 2 and --min_contig_length 200 were used to reduce memory footprint and discard contigs shorter than 200 bp, respectively. In all other respects, default parameters were used. After Trinity assembly, redundant contigs with over 99 % identity were eliminated using CD-HIT-EST [27, 28].

#### Reference-based re-assembly

After *de novo* assembly by Trinity, reference-based re-assembly was performed as described in detail in previous publications [12, 13, 15]. The annotated *Ae. aegypti* protein sequences (AaegL3.3, accessed on October 8, 2014 from VectorBase [29]) were used as the protein reference for the reference-based re-assembly, in order to provide a consistent comparison with TransPS. The *Ae. aegypti* genome scaffolds (AaegL3) and gene annotations (AaegL3.3.gff3) used as the genomic reference were downloaded from Vectorbase [29]. Custom Perl scripts for reference-based re-assembly are available upon request.

#### Transcriptome Post-Scaffolding (TransPS)

After *de novo* assembly by Trinity, TransPS re-assembly [14] was performed using default parameters according to the authors’ instructions. The *Ae. aegypti* proteome was selected as the scaffolding reference, because *Ae. aegypti* is in the same sub-genus (*Stegomyia*) as *Ae. albopictus* and the most closely related species with well-documented genomic information. Because TransPS is developed for the assembly of strand-specific RNA libraries (Z. Adelman, pers. comm.), contigs in the reverse complement orientation to the protein references were first transformed to the same orientation as the protein references based on BLASTX results. All correctly oriented contigs were then searched against the protein references using BLASTX. Contigs that matched the protein references (e-value < 1e-06) were re-assembled by TransPS using default parameters.

#### Genome-guided assembly - *Ae. albopictus* reference genome

The genome assembly of *Ae. albopictus* [21] was used as the genomic reference. This assembly is comprised of ~400,000 scaffolds, with an N50 of ~200 kb [21]. The Tuxedo suite [24], including Bowtie, Tophat and Cufflinks, was used for genome-guided assembly. For each dataset, reads from distinct life stages were first separately mapped to the genome by Tophat (--segment-length 50 --no-coverage-search), to minimize the frequencies of SNPs and computational power required [24]. Mapped reads were assembled by Cufflinks (-u -I 500000), and subsequently merged by Cuffmerge. The default parameters were used except in cases specified in parentheses above. To be consistent with the other assembly strategies, only contigs longer than 200 bp were retained. After genome-guided assembly, redundant contigs with over 99 % identity were eliminated using CD-HIT-EST [27, 28].

#### Genome-guided assembly - *Ae. aegypti* reference genome

Using the 180 M dataset, the genome assembly of *Ae. aegypti* [20] was used as the genomic reference. This assembly is comprised of 4,757 scaffolds, with an N50 of ~1.5 Mb (AaegL3, Vectorbase). The Tuxedo suite [24], including Bowtie, Tophat and Cufflinks, was used for genome-guided assembly. Reads from distinct life stages were first separately mapped to the genome by Tophat using recommended procedures (--segment-length 50 --no-coverage-search), assembled by Cufflinks (-u -I 500000), and subsequently merged by Cuffmerge. A reference annotation file for *Ae. aegypti* (AaegL3.3, Vectorbase) was incorporated into Cuffmerge (-g < reference_annotation.gtf>) to guide the assemblies, as researchers would likely take advantage of the existing annotations. Cuffmerge was also performed without reference annotation. The default parameters were used except in cases specified in parentheses above. To be consistent with the other assembly strategies, only contigs longer than 200 bp were retained. After genome-guided assembly, redundant contigs with over 99 % identity were eliminated using CD-HIT-EST [27, 28].

#### Comparison between genome-guided assemblies using two reference genomes

Using the 180 M dataset, genome-guided assemblies using the *Ae. albopictus* and *Ae. aegypti* (with reference annotation) genomes were aligned to both *Ae. albopictus* and *Ae. aegypti* genomic scaffolds via BLASTN (evalue < 1e-6). Custom Perl scripts were used to calculate percent identity and contig coverage from the alignment results.

### Quality metrics

#### Contig number and length distribution

Fewer and longer contigs lead to more accurate and efficient downstream read mapping. This will in turn optimize subsequent genetic analyses, such as differential gene expression analysis, SNP calling, and novel transcript or splice variant discovery. The number of contigs generated by each assembly method was recorded and the contig length distribution was represented by the median contig length. All contigs from *de novo* assemblies (180 M, 360 M, 600 M datasets) were aligned against the *Ae. aegypti* gene models with and without multiple isoforms using BLASTX (evalue < 1e-6). Contigs with greater than or equal to 70 % identity to the gene models and all isoforms were counted and aggregated for each dataset (180 M, 360 M, 600 M). Unannotated contigs from the reference-based re-assemblies were aligned to the *Ae. albopictus* genomic scaffolds using BLASTN (evalue < 1e-6). Custom Perl scripts were used to calculate percent identity, alignment length and contig coverage.

#### Read mapping

A high percentage of reads mapping back to the transcriptome assembly is desirable for accurate downstream analyses, such as differential gene expression analysis, SNP calling, and novel transcript or splice variant discovery. This is because more reads mapped back to the assembly will result in increased statistical power for performing these analyses. Paired-end reads from each dataset and assembly method were mapped back to the corresponding assembly using Bowtie 2 ([30]; with -N 1 and insert length range specified). The percentage of total reads that mapped back and reads that uniquely mapped back to the assemblies was recorded.

#### Gene model representation

Measures of gene model representation described below include the number of gene models identified in the transcriptome, percent identity between the contigs and reference gene models, and percent reference coverage by the contigs.

#### Number of gene models

A higher number of gene models represented in the transcriptome assembly provides a basis for more comprehensive analysis of the transcriptome under a certain experimental condition. To test the representation of gene models by the transcriptome assemblies, contigs were aligned using BLASTX to the *Ae. aegypti* proteome (AaegL3.3). To provide a second benchmark and minimize potential bias towards reference-based re-assembly and TransPS, the assemblies were also aligned using BLASTX to a non-redundant dipteran protein reference set. This dipteran protein reference set was generated by downloading orthologous protein sequences from *Ae. aegypti*, *Culex quinquefasciatus*, *Anopheles gambiae* and *Drosophila melanogaster* from OrthoDB [31], Version 7 (accessed on October 2, 2014), with one single ortholog retained per ortholog group in the order specified above (i.e., order of relatedness to *Ae. albopictus*). The final reference set contained 19,272 protein sequences, and represented a wide range of evolutionary diversity within Diptera with little redundancy. The best BLASTX matches with e-value smaller than 1e-6 were retained (sorted by bitscore and e-value). Custom Perl scripts were used to calculate the number of unique gene models represented in the transcriptome assemblies. In order to identify putative orthologs, reciprocal BLAST was performed for assemblies using the 180 M dataset against the *Ae. aegypti* protein reference set. Assemblies were searched against the reference set using BLASTX (evalue < 1e-6) and the reference set was searched against the assemblies using TBLASTN (evalue < 1e-6). The reference gene model identified as the best match for a contig by BLASTX was designated as a reciprocal best hit (RBH), a potential ortholog, if the contig was identified as the best match for the gene model by TBLASTN. We didn’t include contigs from the genome-guided assembly using the *Ae. aegypti* reference genome because of its bias towards the *Ae. aegypti* genomic scaffolds (see Additional file 1 and results below). We performed reciprocal BLAST for assemblies using the 180 M dataset against the *Ae. aegypti* protein reference set because we have found that the 180 M dataset is adequate for downstream transcriptome analyses (see Results below), and the *Ae. aegypti* and dipteran protein reference sets yield similar results (see Figs. 3 & 4, Table 1 and Additional files 2 & 3).

**Table 1:** Summary statistics for transcriptome assemblies from multiple strategies

Assembly Strategy	Dataset used	# of contigs	Median length (bp)	Median %ID vs. dipteran	Median reference coverage vs. dipteran	Median %ID vs. aegypti	Median reference coverage vs. aegypti
*De novo* assembly	180M^a^	96,980	414	90.95 %	98.37 %	91.32 %	98.38 %
	360 M	141,390	394	90.57 %	98.21 %	91.02 %	98.14 %
	600 M	176,168	385	90.43 %	98.37 %	90.97 %	98.33 %
Reference-based re-assembly	180 M	89,260	399	91.04 %	98.46 %	91.45 %	98.46 %
	360 M	130,168	382	90.70 %	98.22 %	91.15 %	98.26 %
	600 M	162,168	373	90.57 %	98.39 %	91.10 %	98.45 %
Transcriptome Post-Scaffolding	180 M	11,796	1,783	92.18 %	99.40 %	92.57 %	99.40 %
	360 M	12,027	1,854	91.97 %	99.40 %	92.41 %	99.39 %
	600 M	12,117	1,888	91.90 %	99.41 %	92.39 %	99.41 %
Genome-guided assembly using *Ae. albopictus* genome	180 M	43,537	1,091	90.95 %	97.56 %	91.28 %	97.78 %
	360 M	57,524	1,038	90.73 %	97.57 %	91.05 %	97.77 %
	600 M	68,977	1,000	90.56 %	97.76 %	91.03 %	97.93 %
Genome-guided assembly using *Ae. aegypti* genome + RA^b^	180 M	18,999	1,426	100.00 %	100.00 %	100.00 %	100.00 %
Genome-guided assembly using *Ae. aegypti* genome-RA	180 M	13,230	348	100.00 %	42.47 %	100.00 %	42.89 %

^a^dataset representing approximate number of millions of read pairs used in the corresponding assembly strategy

^b^RA refers to reference annotation

The number of gene models identified by each assembly method using the 180 M dataset from the two reference protein sets was visualized as quadruple Venn diagrams, constructed by the VennDiagram package in the R software environment (www.r-project.org). Genome-guided assembly using the *Ae. aegypti* reference genome was not included in this analysis because of the above-mentioned bias. The 360 M and 600 M datasets were not used in this analysis because each assembly method generated similar numbers of gene models across all three datasets (180 M, 360 M, 600 M) for each of the two reference protein sets (see Results below). The unique gene models identified by *de novo* and genome-guided assemblies were tested for enrichment of specific Gene Ontology (GO) terms and KEGG pathways using the Bioconductor GOseq package [32] in R. GO terms for the dipteran gene models were obtained from Ensembl Metazoa [33]. KEGG pathway assignments for the dipteran organisms in the two reference protein sets were obtained from KEGG API [34]. Because GO terms and KEGG pathways only correspond to genes rather than specific isoforms, isoforms were consolidated within each gene model in the enrichment analyses.

#### Percent identity

A higher median percent identity (PID) indicates higher confidence in annotating the *Ae. albopictus* contigs as putative orthologs of the reference gene models, which is important for interpreting downstream analyses. Custom Perl scripts were used to calculate the PID between contigs and their best BLASTX matches.

#### Reference coverage

A high coverage of the reference gene models by assembled contigs indicates that the annotated gene models are well represented in the *Ae. albopictus* transcriptome, which leads to high confidence in accurate subsequent genetic analyses. Custom Perl scripts were used to calculate the percent coverage for the protein reference in each match (i.e., the proportion of the reference represented in a match).

## Results

Due to the substantial computational requirements of performing transcriptome assembly with some of the methods considered in this study and the large RNA-Seq datasets we analzyed, it was only feasible to perform one assembly for each method and dataset. Therefore, we discuss the absolute magnitude of clear differences and/or high similarities between the assembly methods and between datasets (180 M, 360 M, 600 M) within assembly methods.

### Contig number

Consistently across all three datasets, *de novo* assembly produced the largest numbers of contigs, followed closely by reference-based re-assembly. Genome-guided assembly using the *Ae. albopictus* reference genome produced less than half the number of contigs produced by *de novo* assembly. Genome-guided assembly using the 180 M dataset and the *Ae. aegypti* genome produced similar number of contigs relative to TransPS, but fewer than other assembly methods (Table 1). TransPS produced the smallest numbers of contigs, with over 87 % reduction in the number of contigs relative to *de novo* assembly (Table 1). Consistently across all assembly strategies performed using three datasets, increasing the amount of input read pairs used in the transcriptome assembly led to an almost two-fold increase in the number of contigs generated, except for TransPS, where the increase was minimal (Table 1). The number of contigs aligned to annotated gene models and isoforms did not increase substantially with increasing number of read pairs in the datasets (see Additional file 4). More than 84 % of the unannotated contigs aligned to the *Ae. albopictus* genomic scaffolds, with high median percent identity (~93 %) and contig coverage (>97 %) by the genomic scaffolds, though with short median alignment length (255–258 bp, see Additional file 4).

### Contig length distribution

With reference annotation, genome-guided assembly using the *Ae. aegypti* genome generated contigs longer than any other assembly method, except for TransPS (Table 1). Without reference annotation, genome-guided assembly using the *Ae. aegypti* reference genome generated the shortest contigs (Table 1). Consistently across three datasets among all other assembly methods, *de novo* assembly and reference-based re-assembly generated similar and the smallest median contig lengths. Genome-guided assembly using the *Ae. albopictus* reference genome generated median contig lengths more than twice as long as those by *de novo* assembly and reference-based re-assembly. Finally, the TransPS assembly generated the greatest median contig length with a more than four-fold increase relative to *de novo* assembly and reference-based re-assembly (Table 1). Within each assembly strategy performed using three datasets, increasing the amount of read pairs used in the transcriptome assembly slightly decreased the median contig length, with the exception of TransPS, which generated slightly greater median contig lengths with more input read pairs (Table 1).

### Paired-end read mapping

**Fig. 2:**
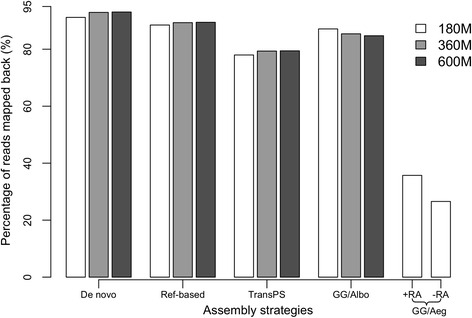
Percentage of paired-end reads mapping back to the assembly. Datasets (180 M, 360 M, 600 M) and assembly strategies as described in Fig. 1, GG/Albo refers to genome-guided assembly using the *Ae. albopictus* reference genome and GG/Aeg refers to genome-guided assembly using the *Ae. aegypti* reference genome. +RA refers to genome-guided assembly using the *Ae. aegypti* genome with reference annotation, and -RA refers to genome-guided assembly using the *Ae. aegypti* genome without reference annotation

Consistently across all three datasets, *de novo* assembly, reference-based re-assembly and genome-guided assembly using the *Ae. albopictus* reference genome produced a similar percentage of paired-end reads mapped back, ranging from 84.73 to 93.06 % (Fig. 2). Less than 80 % of the paired-end reads mapped back to the TransPS assemblies (Fig. 2). Genome-guided assembly using the *Ae. aegypti* reference genome had the lowest percentage of reads mapped back (<40 %, Fig. 2). When considering uniquely mapped reads, *de novo* assembly and reference-based re-assembly had a similar percentage of reads uniquely mapped back and TransPS had a slightly higher percentage of reads uniquely mapped back (all above 70 %, see Additional file 5). Genome-guided assembly using the *Ae. albopictus* reference genome had a much lower percentage of reads uniquely mapped back (<50 %) and genome-guided assembly using the *Ae. aegypti* reference genome had the lowest percentage of reads uniquely mapped back (<8 %, see Additional file 5). Within each assembly strategy performed using three datasets, increasing the amount of read pairs used in the transcriptome assembly did not have a large or consistent effect on the read mapping percentage.

### Number of gene models

**Fig. 3:**
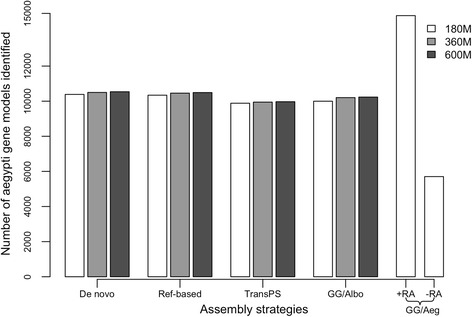
Number of gene models identified from the *Ae. aegypti* reference protein set in all assemblies. Datasets and assembly strategies as in Fig. 2

For all datasets, most assemblies produced similar numbers of gene models from both *Ae. aegypti* and dipteran protein reference sets (see Fig. 3 and Additional file 2), with the number of dipteran gene models generated by TransPS assemblies slightly lower than the other methods (~8,500 vs. >9,100). The exceptions were that genome-guided assembly using the *Ae. aegypti* reference genome with reference annotation produced the highest numbers of both *Ae. aegypti* and dipteran gene models and that genome-guided assembly using the *Ae. aegypti* reference genome without reference annotation produced the lowest numbers of both *Ae. aegypti* and dipteran gene models (see Fig. 3 and Additional file 2). Within each assembly strategy performed using three datasets, increasing the amount of input read pairs led to a very slight increase in the number of *Ae. aegypti* gene models represented in the assembly (~200, Fig. 3), although increased input read pairs did not have an apparent effect on the number of dipteran gene models represented in the assembly (see Additional file 2). The number of gene models discovered from the dipteran protein reference set was smaller than that from the *Ae. aegypti* protein reference set, which is expected due to the closer relationship of *Ae. albopictus* to *Ae. aegypti* relative to the other species in the dipteran protein reference set. Using the 180 M dataset, TransPS generated the highest percentage of RBHs, and the largest number of potential orthologs, amongst all assembly methods, the rest of which performed similarly (see Additional file 6). Genome-guided assembly using the *Ae. albopictus* reference genome had the highest median alignment length when searched against the *Ae. aegypti* protein reference set (see Additional file 6). The protein reference set had the highest median alignment length when searched against the TransPS assembly (see Additional file 6).

**Fig. 4:**
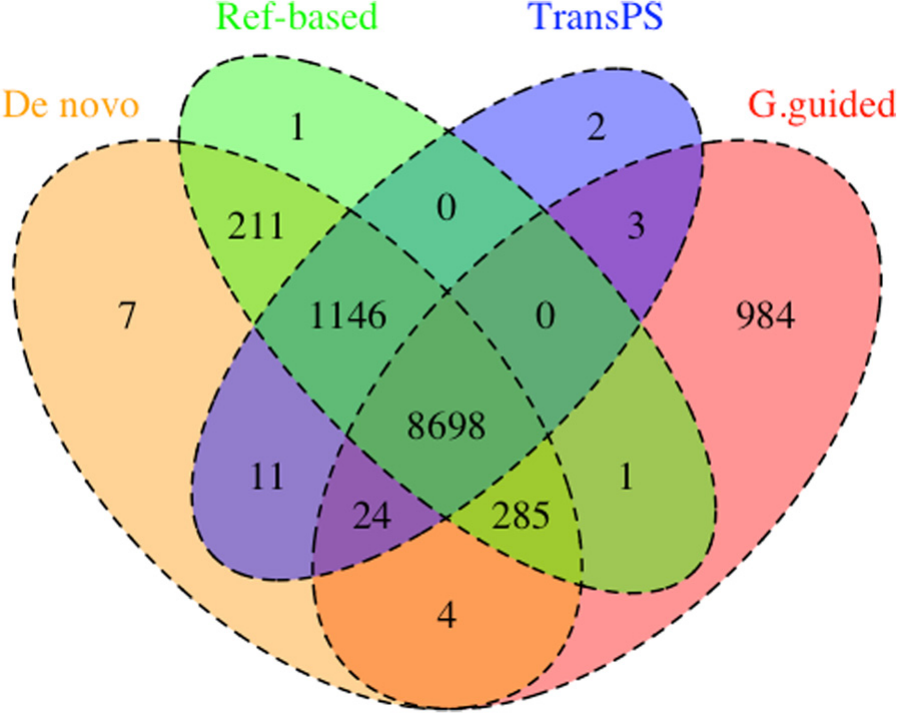
Intersection of *Ae. aegypti* gene models identified by all assembly strategies using the 180 M dataset. Assembly strategies as in Fig. 2, except that G.guided refers to genome-guided assembly using the *Ae. albopictus* reference genome

Using the 180 M dataset, reference-based re-assembly and TransPS identified the same subsets of gene models identified by *de novo* assembly, with very few exceptions (see Fig. 4 and Additional file 3). This result is expected because these two methods are both re-assembly strategies performed after *de novo* assembly. Also using the 180 M dataset, the *de novo* assembly and genome-guided assembly using the *Ae. albopictus* reference genome identified 1,375 and 988 unique gene models relative to each other from the *Ae. aegypti* reference protein set, respectively (Fig. 4). Finally, the *de novo* assembly and genome-guided assembly using the *Ae. albopictus* reference genome identified 1,036 and 935 unique gene models relative to each other from the dipteran reference protein set, respectively, using the 180 M dataset (see Additional file 3). In all cases, the unique gene models were not enriched for specific GO terms or KEGG pathways (all FDR-corrected *p* values are greater than or equal to 0.11).

### Percent identity

Genome-guided assembly using the *Ae. aegypti* reference genome generated contigs with the perfect (100 %) median percent identity (PID) to corresponding *Ae. aegypti* and dipteran gene models (Table 1). Consistently across three datasets among all other assembly methods, TransPS generated contigs with the highest median PID to corresponding *Ae. aegypti* and dipteran gene models, followed closely by reference-based re-assembly, *de novo* assembly and genome-guided assembly using the *Ae. albopictus* reference genome, the latter three with similar PID values (Table 1). Within each assembly strategy performed using three datasets, increasing the amount of read pairs used in the transcriptome assembly decreased median PID to both *Ae. aegypti* and dipteran gene models.

### Reference coverage

Median reference coverage by *Ae. albopictus* contigs for both *Ae. aegypti* and dipteran gene models was high (most above 97.50 %) and similar across most transcriptome assembly strategies using each dataset (Table 1). Genome-guided assembly using the *Ae. aegypti* reference genome with reference annotation produced the highest median reference coverage (100 %). The exception was that genome-guided assembly using the *Ae. aegypti* reference genome without reference annotation produced the lowest median reference coverage (below 50 %).

### Genome-guided assembly - *Ae. albopictus* genome *versus Ae. aegypti* genome

Contigs from the genome-guided assembly using the *Ae. albopictus* reference genome resembled the *Ae. albopictus* genomic scaffolds (median percent identity = 100 %, median contig coverage = 100 %), rather than the *Ae. aegypti* genomic scaffolds (median percent identity = 78.34 %, median contig coverage = 76.02 %, see Additional file 1A). On the contrary, with reference annotation, contigs from the genome-guided assembly using the *Ae. aegypti* reference genome resembled the *Ae. aegypti* genomic scaffolds (median percent identity = 100 %, median contig coverage = 100 %), rather than the *Ae. albopictus* genomic scaffolds (median percent identity = 80.68 %, median contig coverage = 86.90 %, see Additional file 1B). This result likely indicates the presence of ancestral polymorphism in the *Ae. albopictus* genome after 71.4 million years of divergence from *Ae. aegypti* [21] and emphasizes the limitation of using a closely related reference genome for guiding transcriptome assembly with the Tuxedo suite workflow.

## Discussion

Transcriptome assembly is a crucial first step for transcriptome analyses using RNA-Seq data. With the rapid development in the NGS technologies, genomic and transcriptomic resources have accumulated rapidly for a wide range of organisms. However, deciding on the optimal transcriptome assembly strategy for non-model organisms remains a challenge, especially with the large amount of data generated by NGS. This study is the first to compare *de novo* assembly with genome-guided assembly, reference-based re-assembly and TransPS, the latter two methods representing transcriptome re-assembly using protein sequences from closely related species. This study is also one of the first to compare genome-guided assembly using a reference genome from the focal study organism and a reference genome from a closely related species. Our analysis takes advantage of three datasets with increasing numbers of Illumina paired-end reads.

A previous study [35] used genomic contigs from the same species or genomic scaffolds from a closely related species (90–95 % identity) as a scaffold to re-assemble *de novo* contigs generated from RNA-Seq reads. Results from this study [35] showed that genomic sequences from the same species or closely related species can improve the representation of full-length reference transcripts by the *de novo* transcriptome assembly. However, high coverage genomic sequences are often not available together with RNA-Seq reads, as high coverage whole genome sequencing is still expensive, especially for species with a large genome. In addition, genomic resources from closely related species with higher than 90 % identity are relatively rare. In contrast, this study used protein sequences from a more distantly related species (*Ae. aegypti*, in the same sub-genus, *Stegomyia*, as *Ae. albopictus*) as the reference scaffolds. This study also considered genomic scaffolds from *Ae. aegypti* with 80–85 % identity at the nucleotide level relative to the comparison noted above. Thus, both re-assembly approaches used in this study (reference-based re-assembly and TransPS) are likely to apply to a wide range of organisms. Although the optimal assembly strategy will depend upon the purpose and downstream analyses of the specific study, our analysis provides general guidance that is likely to be useful to researchers conducting transcriptome analyses in non-model organisms.

### *De novo* assembly - Trinity

Trinity, which has been optimized for runtime performance [36], has been demonstrated to be an effective *de novo* transcriptome assembler [23, 37, 38]. Trinity *de novo* assembly performed similarly to genome-guided assembly using the *Ae. albopictus* reference genome in terms of percent read mapping back to the assembly and gene model representation, though not in terms of contig number or length distribution. One of the advantages of *de novo* assembly is that gene models can be assembled that may not be assembled by a genome-guided method if the reads cannot be mapped back to the current genomic scaffolds due to improper scaffold assembly (see Fig. 4 and Additional file 3). It is straightforward and fast (24–48 h) to perform the Trinity assembly pipeline using high quality RNA-Seq reads, though it typically requires a large amount of memory (up to 256GB of RAM in this study). Thus, Trinity will usually require a high performance computing cluster or the cloud environment. Trinity incorporates all reads simultaneously without the need to separately perform assemblies of reads from different life stages or environmental conditions. The assemblies generated by the Trinity *de novo* method had high read mapping percentage, and represented a substantial number of gene models with high median PID and coverage. Identifying more total gene models, including paralogs, can be more beneficial for downstream differential gene expression and enrichment analyses, because most paralogs have similar biological functions and are included in gene ontology and KEGG pathways. Trinity assemblies had slightly lower unique mapping rates than TransPS assemblies, but because read mapping programs, such as RSEM [39], take into account reads mapped to multiple gene models, total read mapping rates are more informative than unique mapping rates for most downstream analyses. Therefore, the Trinity assemblies would be suitable for subsequent differential gene expression analysis, SNP discovery in coding regions, and also novel transcript or splice variant discovery. However, the Trinity assemblies contain more redundancy relative to assemblies generated by genome-guided and TransPS methods. Redundant contigs represent highly similar sequences corresponding to the same reference and are in part due to the fact that RNA-Seq was performed on mRNAs from tissue samples that were pooled from multiple individuals, a common practice in RNA-Seq studies. The increased redundancy of the Trinity assemblies is reflected in the larger number of contigs, but similar number and coverage of reference gene models relative to the other assembly methods. The redundancy within the *de novo* assembly increased with increasing amount of input read pairs, primarily due to the increase in unannotated rather than annotated contigs (see Additional file 4). These unannotated contigs align to unannotated protein coding sequences and non-coding sequences, including 5′ and 3′ untranslated regions.

### Reference-based re-assembly

Our previous study [15] has demonstrated that reference-based re-assembly can improve *de novo* assembly in terms of contig number and length distribution. However, in the current study, reference-based re-assembly performed similarly to *de novo* assembly using Trinity (Table 1). There are two likely reasons for this result. First, different versions of Trinity were used in the previous and the current studies, and the latter is an improved version. Second, our previous study only used short RNA-Seq reads from the adult stage for the *de novo* assembly, which were then combined with previous contigs from other life stages in a reference-based re-assembly, including contigs assembled from 454 sequencing reads. However, the current study assembled short Illumina RNA-Seq reads from all life stages into a *de novo* assembly. The contigs from this *de novo* assembly were then used in the reference-based re-assembly, but were not combined with any previously assembled contigs. The advantage of reference-based re-assembly is that it can combine contigs from previous studies under different environmental conditions using different sequencing platforms [12, 13, 15], which makes the re-assembly less computationally demanding and more flexible. Furthermore, reference-based re-assembly can incorporate both protein and genomic sequences from closely related species, maximizing the information provided by reference scaffolding. However, reference-based re-assembly is more complicated to perform than the straightforward Trinity *de novo* assembly pipeline because it involves using several custom Perl scripts for the re-assembly, generally taking approximately 2 weeks to complete after *de novo* assembly.

### TransPS

In the current study, we used a reference proteome from *Ae. aegypti*. As mentioned above, *Ae. aegypti* is in the same sub-genus (*Stegomyia*) as *Ae. albopictus*. This is similar to the level of divergence between one of the organisms (*Ixodes ricinus*) and its reference (*I. scapularis*) used in the original study [14]. TransPS reduced the number of contigs by more than 87 %, a result similar to the original description of this method [14]. TransPS also produced the longest contigs with more than a four-fold increase in the median contig length relative to *de novo* assembly and reference-based re-assembly (Table 1), with minimal redundancy. Thus, the TransPS assemblies had the lowest number of contigs per gene model among all four assembly methods. Moreover, the median coverage for reference gene models by TransPS assemblies was the highest amongst all methods (>99 %), except for the genome-guided assembly using the *Ae. aegypti* genome with reference annotation (Table 1), indicating that TransPS assemblies had almost complete representation for the reference coding sequences. TransPS assembly also had the highest percentage of reciprocal best hits (RBHs, see Additional file 6). Out of 17,158 *Ae. aegypti* gene models, 3,952 are at least 70 % identical to other gene models (not isoforms), with median percent identity of 97.88 % and median coverage of 99.65 %. Because TransPS generated the longest contigs (Table 1) by also merging contigs with non-overlapping alignments to the same gene model, the TransPS contigs can better distinguish small differences between highly similar gene models, thereby identifying the most RBHs (see Additional file 6). This advantage is reflected in the longest median alignment length when searching the reference set against the assemblies (TBLASTN), not when searching the assemblies against the reference set (BLASTX, see Additional file 6). However, the overall number of gene models and read mapping percentage of the TransPS assemblies were slightly lower than the other three strategies, which could limit some downstream analyses such as differential gene expression analysis, SNP discovery, or novel transcript and splice variant discovery. This result is likely due to the algorithm used by TransPS to re-assemble the *de novo* assembly via protein sequences from a closely related species, without the untranslated regions or non-coding sequences in the genomic scaffolds utilized by the reference-based re-assembly or genome-guided assembly. On the other hand, because TransPS had the highest percentage of reads uniquely mapped back, which enables accurate identification of orthologs, TransPS may be preferable for other downstream analyses, such as phylogenomic studies. A few thousand complete and orthologous genes from each transcriptome will be sufficient to construct a phylogeny with high confidence (e.g., [7]). Identifying the largest number of potential orthologs via reciprocal BLAST further strengthens the advantage of using TransPS for phylogenomic studies. As described in the Methods section, TransPS is developed specifically for assembly of strand-specific libraries, and therefore extra steps are required if the initial assembly (prior to scaffold re-assembly) is generated from non-strand-specific sequencing reads. While the TransPS script took an hour to finish, generating the necessary BLASTX outputs and the extra steps necessary to handle non-strand-specific sequencing reads took approximately 2 weeks to complete.

### Genome-guided assembly - Cufflinks: *Ae. albopictus* reference genome

The number and reference coverage of gene models generated by the genome-guided method using the *Ae. albopictus* reference genome were very similar to those produced by *de novo* assembly and reference-based re-assembly. However, genome-guided assemblies using the *Ae. albopictus* reference genome produced less than half of the number of contigs generated by *de novo* assembly and reference-based re-assembly (Table 1), indicating that this assembly produced lower redundancy relative to these two methods. In addition, genome-guided assemblies using the *Ae. albopictus* reference genome produced median contig lengths more than twice as long as those produced by *de novo* assembly and reference-based re-assembly (Table 1). Furthermore, genome-guided assembly is much less computationally demanding than *de novo* assembly. Genome-guided assembly using Cufflinks requires the most computational resources during read mapping to the genome. In this study, up to 72GB of RAM was used for the read mapping using TopHat and up to 36GB of RAM was used for the Cufflinks genome-guided assembly. It took approximately 1 week to complete read mapping and assembly using the pipeline. Another advantage of genome-guided assembly is that transcripts with low sequencing coverage can be assembled if the reads can be mapped to the genomic scaffolds, which can lead to unique gene models assembled relative to *de novo* assembly (see Fig. 4 and Additional file 3). However, genome-guided assembly using the *Ae. albopictus* reference genome performed similarly to those generated by *de novo* assembly and reference-based re-assembly in terms of percent read mapping back to the assembly and gene model representation, and had the lowest unique mapping rates. This is likely because of the fact that the current *Ae. albopictus* genome assembly is relatively fragmented, largely due to its huge genome size and high composition of repetitive elements in the genome [21]. However, the fragmented assembly of the *Ae. albopictus* genome (~400,000 scaffolds with an N50 of ~200 kb) is similar to other insect genomes. For example, the sand fly genome, *Phlebotomus papatasi*, has 106,826 scaffolds with an N50 of 27,956 bp [3]. Therefore, our current results are likely relevant to a wide range of organisms with similarly fragmented genome assemblies. Genome-guided assemblies can be suitable for differential gene expression analysis and SNP discovery, but may be less desirable for novel transcript and splice variant discovery, because only reads aligned to the known splice junctions can be assembled.

### Genome-guided assembly - Cufflinks: *Ae. aegypti* reference genome

*Ae. aegypti* is a closely related species in the same sub-genus (*Stegomyia*) as *Ae. albopictus. Ae. aegypti* has well-annotated genomic resources and a much less fragmented genome assembly, which helps to identify more complete gene models (see Fig. 3 and Additional file 2). However, using the *Ae. aegypti* rather than the more fragmented *Ae. albopictus* genome to guide transcriptome assembly can be problematic. Genome-guided assembly using the *Ae. aegypti* reference genome produced contigs that resemble the *Ae. aegypti* genomic scaffolds, rather than the *Ae. albopictus* genomic scaffolds (see Additional file 1). This result is reflected by the low percentage of reads mapped back, perfect median percent identity and reference coverage values of contigs from the genome-guided assembly using the *Ae. aegypti* genome with reference annotation (see Table 1 and Additional file 5).

### Combination of *de novo* and genome-guided assemblies

Assemblies generated by the *de novo* and genome-guided methods identified a small number of unique gene models relative to each other, which is consistent with a previous study [40]. Therefore, it might be beneficial to combine genome-guided and *de novo* assemblies, as proposed previously [10]. When the genome reference is not well assembled, *de novo* assembly should be performed first, followed by aligning contigs from the *de novo* assembly to the genome reference in order to extend and scaffold the contigs [10]. However, if the annotation of the genome is not complete, additional annotation based on orthologs will still be necessary. As a proof of concept, we combined *de novo* assembly and genome-guided assembly using the *Ae. albopictus* reference genome for the 180 M dataset. The combined assembly encompassed almost all *Ae. aegypti* and dipteran gene models from the two separate assemblies (see Fig. 4 and Additional file 3). This result indicates that combining the *de novo* and genome-guided assemblies can generate a more comprehensive transcriptome assembly in terms of number of gene models identified.

### Effect of the number of reads on assembly quality

The quantity of input read pairs increased the redundancy within the assembly, and decreased both median contig length and PID, whereas it did not have a clear effect on other quality metrics, i.e., percent read mapping and gene model representation. This is likely due to the increasing number of mismatches in the larger datasets caused by SNPs from the pooled tissue samples and/or sequencing errors. These mismatches caused difficulties for the assembly programs to consolidate highly similar contigs. This in turn increased the number of contigs generated (increased redundancy with similar number of gene models and reference coverage), and reduced both the median contig length and PID. Therefore, it will generally be beneficial and cost-effective to use fewer reads (i.e., the 180 M dataset). The exception was TransPS, which generated slightly longer contigs with more input read pairs (Table 1). This is likely because the algorithm used in TransPS that assembles contigs that have non-overlapping matches to the same reference, thereby creating longer contigs [14].

## Conclusions

Our results emphasize the efficacy of *de novo* transcriptome assembly using high quality Illumina RNA-Seq reads and the importance of genome assembly and annotation for genome-guided assembly. Despite generating a more fragmented transcriptome assembly, *de novo* assembly performed similarly to genome-guided assembly using the *Ae. albopictus* reference genome in terms of read mapping and gene model representation. With high quality reads, *de novo* assembly can prove to be effective for organisms without a genome sequence, or for organisms with a fragmented genome assembly relative to traditional model organisms. A recent study points out that even with well curated human and worm genomes, *de novo* assembly still performs similarly to genome-guided assembly in terms of sensitivity in constructing the transcriptome using simulated data [41]. Previous studies have demonstrated a better performance by genome-guided assembly than *de novo* assembly [17, 35]. However, this study showed that, if the genome assembly is fragmented and/or genome annotation is incomplete, *de novo* assembly can perform similarly to or even outperform genome-guided assembly in terms of read mapping back to the assembly and gene model representation. Researchers need to be cautious when using a closely related reference genome to guide transcriptome assembly, because the resulting assembly can be biased towards the closely related genome rather than the focal genome. In our analysis, this result occurred when using a reference genome from the same sub-genus with approximately 70 million years of divergence (see Additional file 1). Reference-based re-assembly performed similarly to *de novo* assembly, whereas TransPS generated the longest contigs with higher reference coverage, least redundancy and largest number of potential orthologs (RBHs). However, TransPS also produced assemblies with the lowest percent read mapping and number of gene models identified. Our results also reveal that the amount of input read pairs tended to reduce the quality of the resulting transcriptome assembly. Thus, 180 M high quality paired-end reads will usually be sufficient to generate a transcriptome assembly appropriate for downstream analyses. The optimal transcriptome assembly strategy is dependent upon intended downstream analyses, but in general, Trinity *de novo* assembly with 180 M high quality read pairs is suitable for most downstream transcriptome analyses, especially for organisms without a genome sequence. If a genome assembly is present, it can be beneficial to combine *de novo* and genome-guided assemblies when the computational resources are available.

## Abbreviations

GO, Gene Ontology; NGS, Next-generation Sequencing; PID, Percent Identity; RBH, Reciprocal Best Hit; RNA-Seq, RNA-Sequencing; SNP, Single Nucleotide Polymorphism; STM, Scaffolding using Translation Mapping; TransPS, Transcriptome Post-Scaffolding.

## Additional files

Additional file 1: Similarity of genome-guided assemblies using the 180 M dataset to reference genome assemblies. Percent identity to and contig coverage by the *Aedes albopictus* or *Aedes aegypti* genomic scaffolds characterized by BLASTN searches (evalue < 1e-6). A: Comparisons between genome-guided assembly using the *Ae. albopictus* genome assembly or the *Ae. aegypti* genome assembly and the *Ae. albopictus* genomic scaffolds. B: Comparisons between genome-guided assembly using the *Ae. albopictus* genome assembly or the *Ae. aegypti* genome assembly and the *Ae. aegpyti* genomic scaffolds. Albopictus G.guided: genome-guided assembly using the *Ae. albopictus* genome; Aegypti G.guided: genome-guided assembly using the *Ae. aegypti* genome with reference annotation; Albopictus scaffolds: genomic scaffolds of *Ae. albopictus*; Aegypti scaffolds: genomic scaffolds of *Ae. aegypti*. (PDF 6 kb) Additional file 2: Number of gene models identified from the dipteran reference protein set in all assemblies. Datasets and assembly strategies as in Fig. 2. (TIF 8440 kb) Additional file 3: Intersection of dipteran gene models identified by all four assembly strategies using the 180 M dataset. Assembly strategies as in Fig. 2, except that G.guided refers to genome-guided assembly using the *Ae. albopictus* reference genome. (TIFF 902 kb) Additional file 4: Alignment to *Ae. aegypti* gene models and *Ae. albopictus* genomic scaffolds by contigs in the *De novo* assemblies and reference-based re-assemblies with increased number of input read pairs. For *De novo* assemblies: dataset used, number of contigs, the total number of contigs supporting *Ae. aegypti* gene models with multiple isoforms, the total number of contigs supporting *Ae. aegypti* gene models with a single isoform and the total number of contigs supporting all isoforms. Support is characterized as the best blastx match (evalue < 1e-6) with percent identity > = 70 %. For unannotated contigs from the reference-based re-assemblies: dataset used, number of contigs, total number of contigs aligning to the *Ae. albopictus* genome (blastn with evalue < 1e-06), median percent identity of the matches, and median alignment length with the median contig coverage in parentheses. (XLSX 40 kb) Additional file 5: Total and unique read mapping percentages. Dataset used in, percentage of total reads and percentage of reads uniquely mapped back to the transcriptome assemblies generated in this study. (XLSX 34 kb) Additional file 6: Number and percentage of gene models identified by reciprocal BLAST and median alignment length. For various transcriptome assembly methods: Dataset used, number of *Ae. aegypti* gene models identified by BLASTX, percentage of *Ae. aegypti* gene models identified by reciprocal blast (BLASTX and TBLASTN), median alignment length from BLASTX and TBLASTN. All BLAST matches have e-value < 1e-6 and percent identity > = 70 %. (XLSX 30 kb)
