# Supplementary material for: Comparative performance of transcriptome assembly methods for non-model organisms
Source: BMC Genomics. 2016 Jul 27;17:523. doi: 10.1186/s12864-016-2923-8 (PMC4964045; doi:10.1186/s12864-016-2923-8)

A. Percent identity and contig coverage against the Aedes albopictus genomic scaffolds

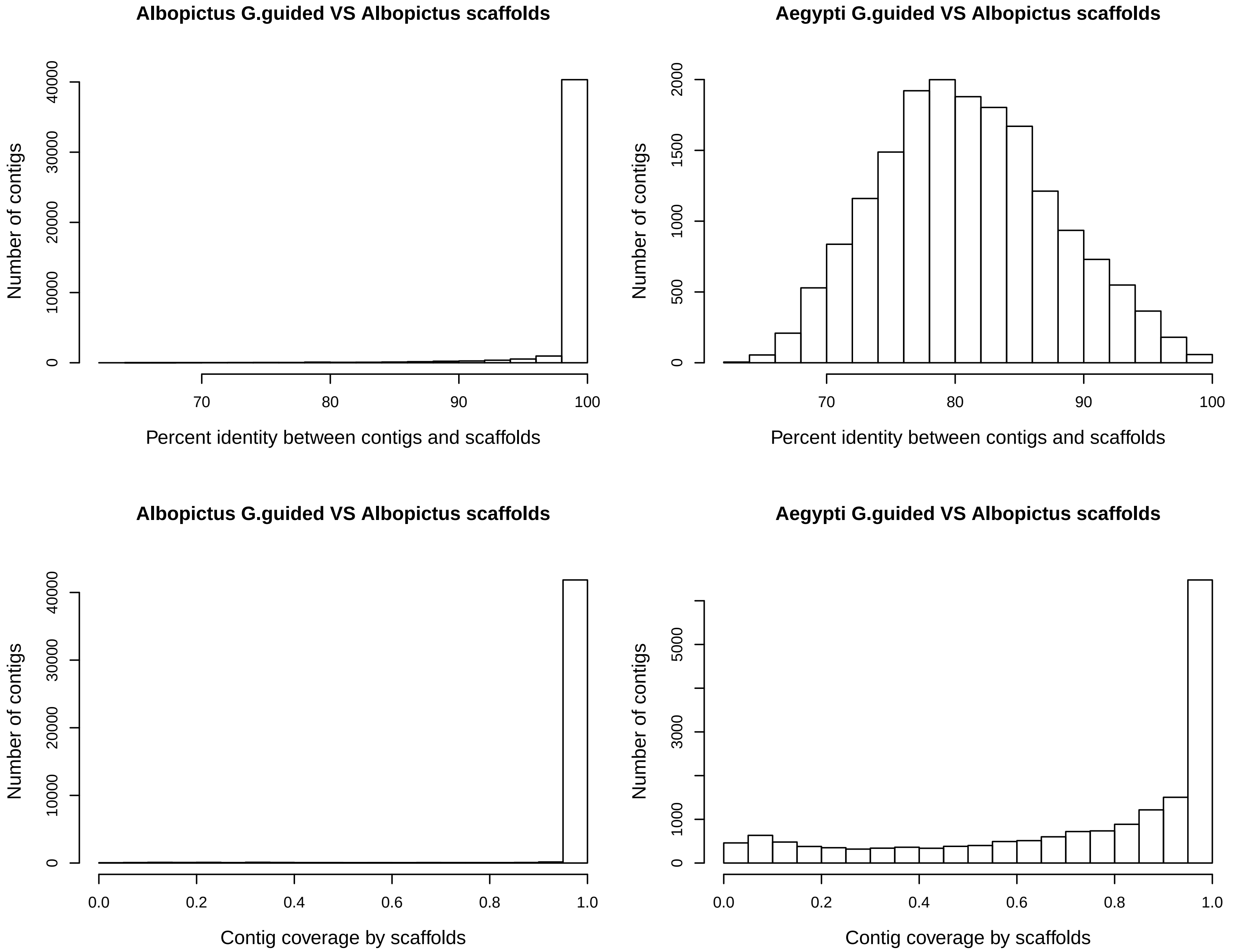

B. Percent identity and contig coverage against the Aedes aegypti genomic scaffolds

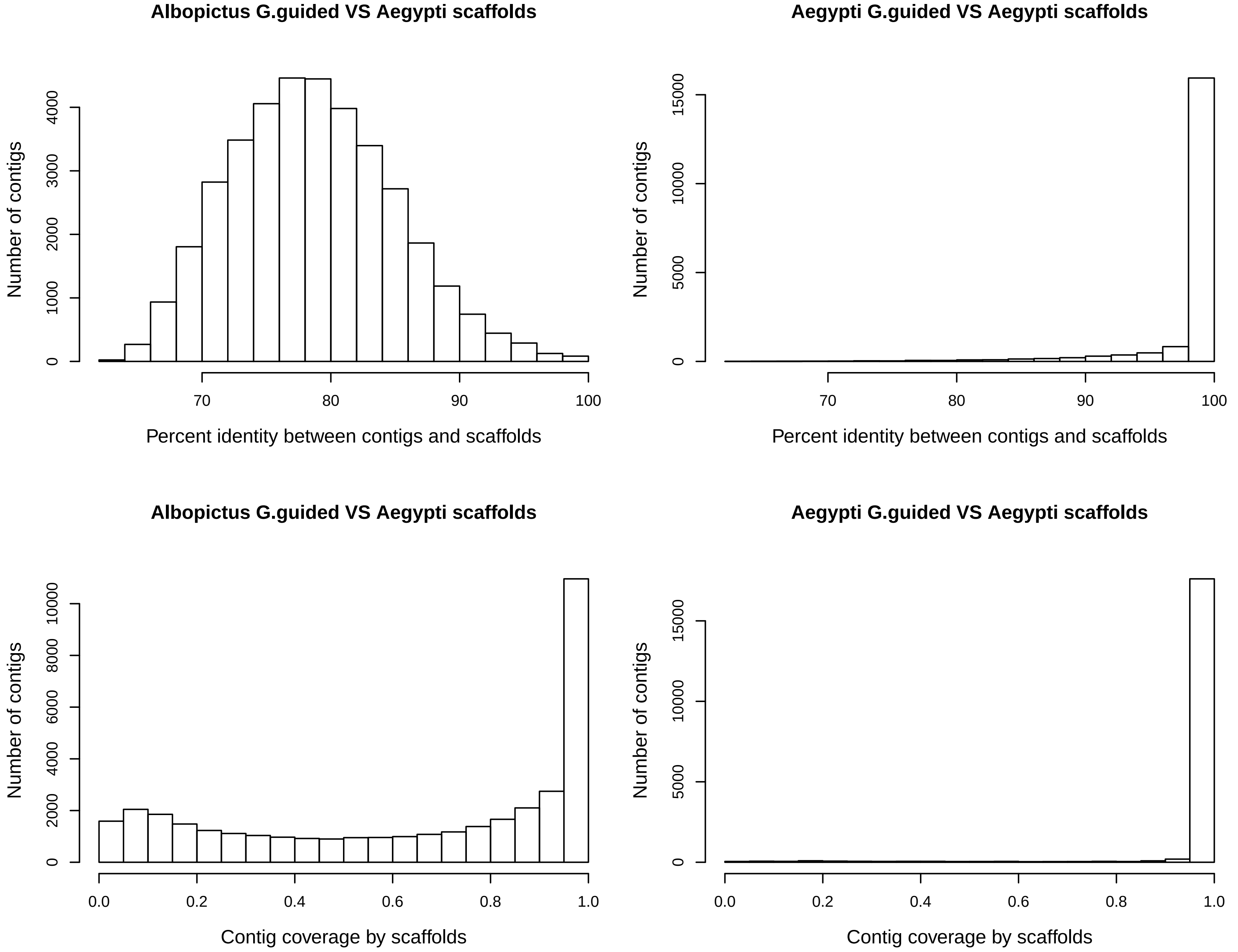

Supplement: Additional file 1: — Similarity of genome-guided assemblies using the 180 M dataset to reference genome assemblies. Percent identity to and contig coverage by the Aedes albopictus or Aedes aegypti genomic scaffolds characterized by BLASTN searches (evalue < 1e-6). A: Comparisons between genome-guided assembly using the Ae. albopictus genome assembly or the Ae. aegypti genome assembly and the Ae. albopictus genomic scaffolds. B: Comparisons between genome-guided assembly using the Ae. albopictus genome assembly or the Ae. aegypti genome assembly and the Ae. aegpyti genomic scaffolds. Albopictus G.guided: genome-guided assembly using the Ae. albopictus genome; Aegypti G.guided: genome-guided assembly using the Ae. aegypti genome with reference annotation; Albopictus scaffolds: genomic scaffolds of Ae. albopictus; Aegypti scaffolds: genomic scaffolds of Ae. aegypti. (PDF 6 kb) [file 12864_2016_2923_MOESM1_ESM.pdf]
