# Supplementary figures and images for: Comparative performance of transcriptome assembly methods for non-model organisms
Source: BMC Genomics. 2016 Jul 27;17:523. doi: 10.1186/s12864-016-2923-8 (PMC4964045; doi:10.1186/s12864-016-2923-8)

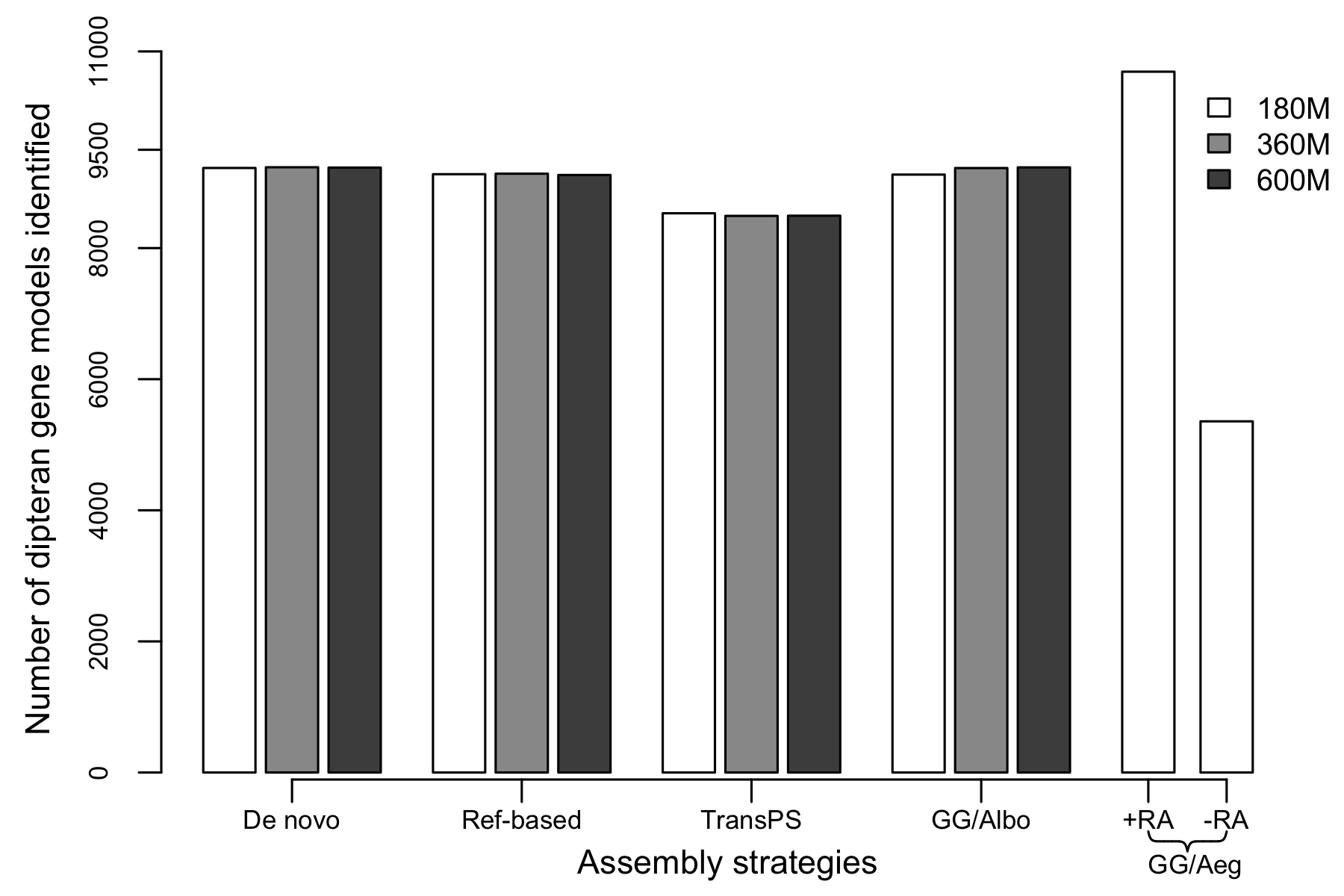

Supplement: Additional file 2: — Number of gene models identified from the dipteran reference protein set in all assemblies. Datasets and assembly strategies as in Fig. 2. (TIF 8440 kb) [file 12864_2016_2923_MOESM2_ESM.tif]

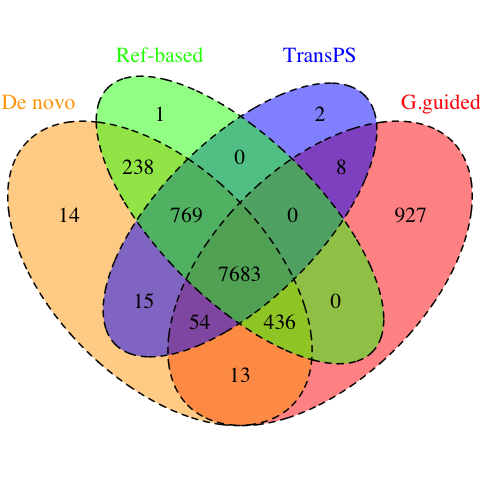

Supplement: Additional file 3: — Intersection of dipteran gene models identified by all four assembly strategies using the 180 M dataset. Assembly strategies as in Fig. 2, except that G.guided refers to genome-guided assembly using the Ae. albopictus reference genome. (TIFF 902 kb) [file 12864_2016_2923_MOESM3_ESM.tiff]
